# Innate immune response of rainbow trout erythrocytes to spinycterins expressing a downsized viral fragment of viral haemorrhagic septicaemia virus

**DOI:** 10.1016/j.aquaculture.2023.739303

**Published:** 2023-04-15

**Authors:** S. Puente-Marin, D. Cazorla, V. Chico, J. Coll, M. Ortega-Villaizan

**Affiliations:** a Instituto de Biologia Molecular y Celular, Universidad Miguel Hernández (IBMC-UMH), Elche, Spain; b Instituto de Investigación, Desarrollo e Innovación en Biotecnología Sanitaria de Elche (IDiBE-UMH), Elche, Spain; c Instituto Nacional de Investigación y Tecnología Agrarias y Alimentarias, Dpto. Biotecnología. INIA, crt.Coruña km 7, 20040 Madrid, Spain

**Keywords:** Spinycterin, Erythrocytes, Red blood cells, immune response, Vaccine, Surface display, bacteria, Bacterin, Fish, Virus, VHSV, Anchor

## Abstract

Recent studies have reported on the importance of RBCs in fish responses to viral infections and DNA vaccines. Surface-displaying recombinant bacterins (spinycterins) are a safe and adaptable prototype for viral vaccination of fish and represent an alternative method of aquaculture prophylaxis, since have been reported to enhance fish immune response. We evaluated the innate immune response of rainbow trout (*Oncorhynchus mykiss*) red blood cells (RBCs), head kidney, and spleen to spinycterins expressing a fragment of the glycoprotein G of viral haemorrhagic septicemia virus (VHSV), one of the most devastating world-wide diseases in farmed salmonids. We first selected an immunorelevant downsized viral fragment of VHSV glycoprotein G (frg16_252–450_). Then, spinycterins expressing frg16_252–450_ fused to Nmistic anchor-motif (Nmistic+frg16_252–450_) were compared to spinycterins expressing frg16_252–450_ internally without the anchor motif. Nmistic+frg16_252–450_ spinycterins showed increased attachment to RBCs *in vitro* and modulated the expression of interferon- and antigen presentation–related genes in RBCs *in vitro* and *in vivo*, after intravenous injection. In contrast, the head kidney and spleen of fish injected with frg16_252–450_, but not Nmistic+frg16_252–450_, spinycterins demonstrated upregulation of interferon and antigen-presenting genes. Intravenous injection of Nmistic+frg16_252–450_ spinycterins resulted in a higher innate immune response in RBCs while frg16_252–450_ spinycterins increased the immune response in head kidney and spleen. Although more studies are required to evaluate the practicality of using spinycterins as fish viral vaccines, these results highlight the important contribution of RBCs to the fish innate immune response to antiviral prophylactics.

## Highlights

• We have evaluated the immune response of RBCs to spinycterins expressing frg16_252–450_ inside or fused to Nmistic anchor-motif.
• Nmistic+frg16_252‐450_ spinycterins showed higher attachment to RBCs and modulated the expression of immune-related genes.
• Nmistic+frg16_252‐450_ spinycterins triggered higher innate immune response in RBCs than in head kidney and spleen.

## 1 Introduction

Fish red blood cells (RBCs) are nucleated cells containing organelles in their cytoplasm (Glomski et al., 1992; Sun et al., 2019). Nucleated RBCs participate in the immune system *via* pattern recognition receptors (PRRs) that recognize pathogen-associated molecular patterns (PAMPs); the production of cytokines or antiviral proteins (Morera et al., 2011; Morera and Mackenzie, 2011; Nombela and Ortega-Villaizan, 2018; Chico et al., 2019a); antigen processing and presentation (Nombela et al., 2019; Puente-Marin et al., 2019a); and leukocyte activity modulation (Workenhe et al., 2008). In addition, fish RBCs produce an immune response when infected by viruses that replicate inside RBCs—such as infectious salmon anaemia virus (ISAV) (Workenhe et al., 2008) or piscine orthoreovirus (PRV) (Wessel et al., 2015; Wessel et al., 2018)—or by viruses that do not replicate inside RBCs—such as viral haemorrhagic septicaemia virus (VHSV) (Nombela et al., 2019; Nombela et al., 2017a; Chico et al., 2019b) or infectious pancreatic necrosis virus (IPNV) (Nombela et al., 2017b). Furthermore, and most important for this work, RBCs behave like non-professional phagocytic cells (Neumann et al., 2001; Rowley et al., 1988) or atypical antigen-presenting cells (APCs) (Nombela et al., 2019; Puente-Marin et al., 2019a; Puente-Marin et al., 2018a; Puente-Marin et al., 2018b).

Teleost RBCs can engulf bacteria and latex particles through different pathways (Sun et al., 2019) as well as bacterial inclusion bodies (IB) made of a VHSV(IB^frg16G−VHSV^) fragment and tumour necrosis factor α (IB^TNFα)^, modulating the immune response accordingly (Puente-Marin et al., 2019b). Moreover, rainbow trout RBCs can modulate the expression of immune genes and proteins when transfected with the glycoprotein G of VHSV (GVHSV) DNA vaccine *ex vivo* and can induce interferon-induced GTP-binding protein Mx (*mx*) gene expression and confer anti-VHSV protection in the rainbow trout gonad 2 (RTG-2) cell line (Puente-Marin et al., 2019a; Puente-Marin et al., 2018b). Likewise, GVHSV-transfected RBCs induce upregulation of differentiation markers in a rainbow trout spleen monocyte/macrophage (RTS11) cell line and stimulate proliferation of rainbow trout white blood cells (WBCs) (Puente-Marin et al., 2019a; Puente-Marin et al., 2018b).

Some of these *in vitro* studies have been complemented with *in vivo* experimentation. VHSV-specific IgM has been detected in the serum of rainbow trout intravenously reinfused with autologous RBCs transfected *in vitro* with the GVHSV DNA vaccine (Puente-Marin et al., 2019a). In addition, intravenous injection of IB^frg16G−VHSV^ has been shown to induce the expression of immune-related genes in RBCs (Puente-Marin et al., 2019b). The contributions of RBCs to fish immunity, as well as the large number of cells and rapid and widespread distribution throughout the body, make these cells novel targets for fish vaccination strategies and indicators of the innate immune response.

Among the pathogens that cause significant losses in both wild and cultivated fresh and saltwater fish are viruses of the Rhabdoviridae family. Among them, VHSV is responsible for viral haemorrhagic septicaemia (VHS), a lethal infectious fish disease that affects >50 species of fish and causes high mortality (up to 100% in fingerlings) (World Organization for Animal Health, O, 2019). DNA vaccination has emerged as one of the best strategies to prevent and control viral diseases and has been shown to be effective against VHSV (Lorenzen et al., 1998). However, many issues related to the commercialization of DNA vaccines need to be resolved, and effective DNA vaccines against most fish viruses are not available (Gomez-Casado et al., 2011). The search for new and effective vaccines to improve applicability, safety, and immunogenicity has become a priority in aquaculture.

To overcome the safety problems associated with live attenuated or DNA vaccines, we present an irreversibly DNA-damaged, surface-displaying, recombinant bacteria (spinycterins) that can be engineered for application against a wide range of diseases. The irreversible DNA damage increases the safety of spinycterins and also has the advantage of supporting lyophilisation, thus bypassing the challenge of low temperature requirements for attenuated vaccines. The use of a whole bacteria as a carrier supports adjuvant properties to stimulate the fish immune system (Coll, 2017; Lama et al., 2019). Moreover, the use of prokaryotic expression plasmids to express viral proteins reduces environmental concerns raised by DNA vaccination based on eukaryotic expression plasmids, thus allowing for mass vaccination strategies (Coll, 2017). Thus, spinycterins constitute a safe alternative for fish viral vaccination within the aquaculture sector.

The use of recombinant bacterins expressing a viral antigen on their surface has been previously tested in fish. *Escherichia coli* bacterins expressing cyprinid herpesvirus type 3 linear antigen (CyHV3) induce the production of antibodies against the viral antigen, (Coll, 2017) and *E.coli* expressing viral nervous necrosis virus (VNNV) antigen conferred protection against VNNV challenge (Lama et al., 2019). Recombinant *Aeromonas hydrophila* bacterins expressing an infectious spleen and kidney necrosis virus (ISKNV) antigen induced the production of antibodies against the virus and the bacteria, providing a protective response against subsequent exposure to both pathogens (Fu et al., 2016).

The aim of this work was to study the immune response triggered by a VHSV bacterial-based vaccine (spinycterins) in rainbow trout RBCs, a cell type that appears to play an important role in the immune system. In the present work, we show for the first time that spinycterins can modulate interferon- and antigen presentation–related genes in rainbow trout RBCs, head kidney, and spleen after immunization. Our results suggest that surface expression of the antigen by spinycterins results in a higher innate immune response in RBCs compared to that in head kidney and spleen.

## 2 Methods

### 2.1 Construction of downsized GVHSV fragment and genetic fusion to prokaryotic anchor-motifs

The fragment16 sequence, amino acid residues 252–450 (frg16_252–450_), was derived from the carboxy-terminal part of GVHSV strain 07–71 (Uniprot KB P27662, NCBI GenBank X59148). The fragment contains both integrin receptor RGD binding sites and inducers of *mx* gene expression sites (Encinas et al., 2011a; Chico et al., 2010) and binds to specific anti-VHSV rainbow trout antibodies in fish that have survived VHSV infection (Encinas et al., 2011b). The GVHSV-derived sequence was fused downstream of 2 different bacterial membrane anchor-motif sequences (Table 1) as previously described (Lama et al., 2019), bracketed by an arbitrarily chosen flexible linker (coding for GliSerGlicSer, GSGS). All corresponding synthetically fused DNA sequences (GeneArt) were cloned into the pRSET prokaryotic expression plasmid, which adds 6 histidine tails (polyH) at the C-terminal end. The general formula of the resulting recombinant constructs is H_2_N-anchor-motif + GSGS + frg16_252–450_ + GSGS + polyH-COOH. The purified plasmids were transfected into *E. coli* BL21(DE3) (Sigma-Aldrich) *via* the CaCl_2_ method and grown in Super Broth (SB) medium at 37 °C.

**Table 1:** Anchor-motifs fused to the N-terminus of frg16_252–450_ and resulting total molecular weights.

Name	AccNum	KDa	frg16_252–450_ and anchor reference
none + frg16_252–450_	AY284969	24.0	(Encinas et al., 2011a; Encinas et al., 2011b)
Nmistic + frg16_252–450_	AY874162	27.8	(Roosild et al., 2005; Alves et al., 2017)
YBEL + frg16_252–450_	NP_415176	42.6	(Leviatan et al., 2010)

Most of the anchor motifs were already tested for *E. coli* expression when fused to fragments derived from CyHV-3 herpesvirus (Coll, 2017) and VNNV betanodavirus (Lama et al., 2019). The anchor-motif+GSGS+frg16_252–450_ + GSGS+polyH DNA sequences were designed, synthesized, cloned into pRSET, used to transform *E. coli*, and induced with IPTG to overexpress the recombinant proteins. AccNum, Gene Bank accession numbers. frg16_252–450,_ amino acid residues 252–450 from the G glycoprotein of VHSV (X59148) (Encinas et al., 2011a; Encinas et al., 2011b). KDa, expected molecular weight of the recombinant proteins. N-mistic, 33 N-terminal amino acid anchor-motif from the Mistic gene from *Bacillus subtilis.* YBEL*,* 160–amino acid anchor-motif hydrophilic HTH-type transcriptional regulator DUF1451 family protein from *E. coli.*

### 2.2 Protein expression induction and inactivation of surface-displaying recombinant bacteria

*E. coli* BL21(DE3) recombinant bacteria were grown overnight with strong agitation at 37 °C in 40 mL of autoinductive SB medium (2.4% yeast extract, 0.8% glycerol, 0.9% KHPO_4_, 0.2% KH_2_PO_4_, 4.8% soybean hydrolysate, 0.3% glucose) (Lama et al., 2019) with 100 μg/mL ampicillin (Sigma-Aldrich). Autoinduction was allowed for 2 to 4 days, and the resulting bacteria were washed in phosphate-buffered saline (PBS) (Sigma-Aldrich) and adjusted to a final concentration of 10^10^ colony forming units (cfu)/mL (Lama et al., 2019). To irreversibly damage bacterial DNA, 100 μg/mL ciprofloxacin (Sigma-Aldrich) was added, and cultures were incubated at room temperature for 2 h with agitation (Lama et al., 2019). The resulting spinycterins were washed with PBS and stored in 20% glycerol (Sigma-Aldrich) at −20 °C until use.

**Fig. S1:**
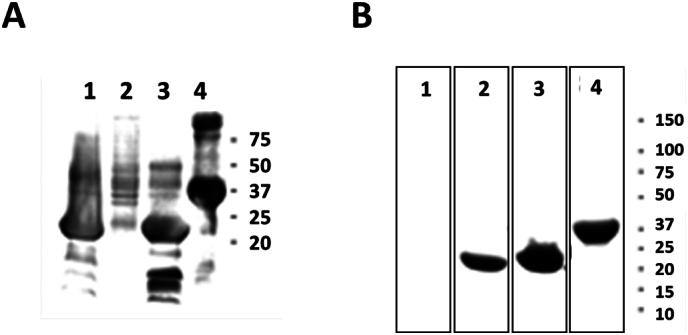
Coomassie-blue staining of anchor-motif + frg16_252–450_ spinycterins. BL21 (DE3) *E. coli* coding for anchor-motif+frg16_252–450_ + polyH recombinant proteins were grown in autoinduction SB medium overnight and killed with ciprofloxacin. Spinycterins were pelleted. A) PAGE analysis of spinycterins after Coomassie blue staining is shown. Numbers to the right of the gels indicate KDa positions of molecular weight markers. The anchor motifs of the spinycterins correspond to lanes as follows: 1, frg16_252–450_; 2, empty plasmid; 3, Nmistic+frg16_252–450_; and 4, YBEL+frg16_252–450_. B) Western blotting. Polyacrylamide gels were transferred to nitrocellulose membranes, stained with anti-polyhistidine monoclonal antibody and peroxidase-labeled anti-rabbit immunoglobulins, and visualized with diaminobenzidine. Numbers to the right of the gels indicate KDa positions of molecular weight markers. The anchor-motifs of the spinycterins correspond to lanes as follows: 1, empty plasmid; 2, frg16_252–450_; 3, Nmistic+frg16_252–450_; and 4, YBEL+frg16_252–450_.

Confirmation of pRSET-anchor+frg16_252–450_ construct-coded protein expression (frg16_252–450_, Nmistic+frg16_252–450_, and YBEL+frg16_252–450_) was performed by polyacrylamide gel electrophoresis (PAGE). Bacterial pellets were incubated at 100 °C for 5 min in buffer containing sodium dodecyl sulphate (SDS) (NZYTech) and ß-mercaptoethanol (Sigma-Aldrich). Proteins were separated in precast gels (4–20% polyacrylamide gel, BioRad). Protein expression was estimated by Coomassie-blue staining (Supplementary Fig. S1A). The proteins separated in precast gels (4–20% polyacrylamide gel) were transferred to nitrocellulose membranes (BioRad) and blocked with dilution buffer (0.5% bovine serum albumin, 5% skim milk, 0.1% Tween-20, 0.01% merthiolate, and 0.005% phenol red in PBS pH 6.7). Nitrocellulose membranes were incubated with mouse anti-polyhistidine antibody and peroxidase-conjugated rabbit anti-mouse antibody (Sigma-Aldrich). Bands were visualized with diaminobenzidine (DAB) stain (Sigma-Aldrich) (Supplementary Fig. S1B).

### 2.3 Maintenance of rainbow trout and culture of RBCs

Rainbow trout (*Oncorhynchus mykiss*) individuals of ∼5–6 cm were obtained from a commercial farm (Piszolla S.L., Cimballa Fish Farm, Zaragoza, Spain), maintained at 14 °C at the University Miguel Hernandez (UMH) facilities in aquaria with biological filters, and fed daily with a commercial diet (Skretting). Fish were acclimatised to laboratory conditions for 2 weeks before the experiments.

During experiments, fish were monitored 2 to 4 times daily. Those with abnormal behaviour were euthanised by tricaine (tricaine methane sulfonate, Sigma-Aldrich) overdose to minimise suffering. Fish were handled in accordance with the National and European guidelines and regulations on the care of laboratory animals. All experiments were performed using protocols approved by the European Union Council Guidelines (86/609/EU). Animal work was approved by the UMH or INIA corresponding Animal Welfare Body and the Research Ethics Committee. All methods were carried out in accordance with the Spanish Royal Decree RD 53/2013 and EU Directive 2010/63/EU for the protection of animals used for research experimentation and other scientific purposes.

### 2.4 RBC culture

Rainbow trout RBCs were obtained from the peripheral blood of fish sacrificed by overexposure to 0.3 g/L tricaine. RBCs were purified by 2 consecutive density gradient centrifugations (7206 g, Ficoll 1.007; Sigma-Aldrich). Purified RBCs were cultured in RPMI-1640 medium (Dutch modification) (Gibco, Thermo Fisher Scientific, Inc.) supplemented with 10% fetal bovine serum (FBS) gamma-irradiated (Cultek), 1 mM pyruvate (Gibco), 2 mM l-glutamine (Gibco), 50 μg/mL gentamicin (Gibco), 2 μg/mL fungizone (Gibco), 100 U/mL penicillin (Sigma-Aldrich), and 100 μg/mL streptomycin (Sigma-Aldrich) at a density of 10^6^ cells/mL and kept at 14 °C until use.

### 2.5 Treatment of RBCs with spinycterins

To assay for spinycterin attachment and interaction capacity, purified RBCs were incubated with frg16_252–450_, Nmistic+frg16_252–450_, and YBEL+frg16_252–450_ spinycterins at a multiplicity of infection (MOI) of 40 spinycterins per RBC in RPMI 10% FBS, 14 °C for 24 h. Spinycterins transformed with the empty pRSET plasmid were included as a control. RBC pellets recovered by centrifugation at 1600 rpm for 5 min were washed with PBS to eliminate unbound spinycterins. The spinycterin-RBC complex was stained with a mouse monoclonal anti-polyhistidine antibody (Sigma-Aldrich) and a rabbit anti-mouse IgG − FITC antibody (Sigma-Aldrich). RBC pellets were washed, and the percentage of fluorescent RBCs were calculated from a total of 10,000 events analysed by flow cytometry in a BD FACS Canto II flow cytometer (BD Biosciences).

Scanning electron microscopy (SEM) micrographs were acquired with field emission SEM (FESEM) (Sigma 300 VP; Carl Zeiss AG, Germany) at the Institute of Research, Development, and Innovation in Healthcare Biotechnology in Elche (IDiBE) to evaluate the interaction and attachment of spinycterins to RBCs. Purified RBCs were incubated with Nmistic+frg16_252–450_ at MOI 40 in RPMI 10% FBS at 14 °C for 24 h. Cell culture medium was removed, and RBCs were washed twice with PBS as indicated above. For SEM micrographs, cells were fixed with 4% paraformaldehyde (PFA) (Sigma-Aldrich) and dried on a carbon conductive tab on top of an aluminium stub, and secondary electron (SE) images were taken. To evaluate the engulfment of spinycterins by RBCs, scanning transmission electron microscopy (STEM-in-SEM) micrographs were taken; RBCs were prepared as previously described (Chico et al., 2018).

To assay the immune response of RBCs to spinycterins *in vitro*, purified RBCs were incubated with the spinycterins expressing frg16_252–450_ or Nmistic+frg16_252–450_ at MOI 40 for 24 h at 14 °C. Spinycterins transformed with the empty pRSET plasmid were used as control. Then, RBCs were washed twice with PBS and the cell pellet stored in TRK lysis buffer at −80 °C for subsequent RNA extraction.

### 2.6 Intravenous injection of rainbow trout with spinycterins

To investigate the effects of spinycterins *in vivo*, juvenile rainbow trout (∼15 g) were anaesthetised with tricaine (40 mg/L) and intravenously injected using insulin syringes (NIPRO) with a 26G needle (BD) in the caudal vein with 5 × 10^8^ cfu spinycterins in a volume of 50 μL PBS. Assuming that the blood of a ∼ 15-g rainbow trout contains ∼1.2 × 10^8^ RBCs, ∼4 spinycterins per RBC (MOI 4) were injected per fish. PBS alone or PBS with spinycterins transformed with the empty pRSET plasmid were injected as controls. At 48 h post-injection, fish were sacrificed by an overdose of tricaine (0.3 g/L). Head kidney, spleen, and RBCs (following purification, as described above) were aseptically recovered and stored in TRK lysis buffer at −80 °C for subsequent RNA extraction.

### 2.7 RNA extraction, cDNA synthesis, qPCR, and gene expression analysis

Head kidney, spleen, and RBC samples were sonicated (Branson 250 Analog Sonifier) for 15 s on ice. E.Z.N.A. Total RNA Kit I (Omega Bio-Tek) was used for RNA purification following the manufacturer's instructions as previously described (Nombela et al., 2017a). RNA was quantified using a NanoDrop 1000 spectrophotometer (Thermo Fisher Scientific, Inc.). A final DNAase treatment with TURBO DNA-free kit (Thermo Fisher Scientific, Inc.) was carried out. RNA was stored at −80 °C until the subsequent synthesis of complementary DNA (cDNA). For cDNA synthesis, the Moloney murine leukaemia virus reverse transcriptase enzyme (M-MLV) (Thermo Fisher Scientific, Inc.) was used as previously described (Chico et al., 2006).

The level of gene expression was analysed by quantitative polymerase chain reaction (qPCR) in a final volume of 20 μL. The reaction mixture of each sample contained 24 ng of cDNA. The qPCR reaction, cycling conditions and gene expression analysis methods were carried out as previously described (Nombela et al., 2017a). Primers and probes are listed in Table 2. Gene expression was analysed by means of the 2 ^−ΔCt^ or 2 ^−ΔΔCt^ (Livak and Schmittgen, 2001), where the *ef1α* gene was used as endogenous control.

**Table 2:** Primers and probes used for qPCR.

*Gene*	Forward primer	Reverse primer	Probe	Reference
*cd83*	TTGGCTGATGATTCTTTCGATATC	TGCTGCCAGGAGACACTTGT	TCCTGCCCAATGTAACGGCTGTTGA	(Ortega-Villaizan et al., 2012)
*ef1α*	ACCCTCCTCTTGGTCGTTTC	TGATGACACCAACAGCAACA	GCTGTGCGTGACATGAGGCA	(Raida and Buchmann, 2007)
*hepcidin*	TCCCGGAGCATTTCAGGTT	GCCCTTGTTGTGACAGCAGTT	AGCCACCTCTCCCTGTGCCGTTG	(Nombela et al., 2017a)
*ifit5*	CCCTGCCCTCATCTTTCTTCT	CCCTCAATGACTCTGACAAGCA	CCAGCTTCGGCCTGTTTCTGTTCCA	(Puente-Marin et al., 2018b)
*il1b*	GCCCCCAACCGCCTTA	CAGTGTTTGCGGCCATCTTA	ACCTTCACCATCCAGCGCCACAA	(Chico et al., 2018)
*il8*	AGAGACACTGAGATCATTGCCAC	CCCTCTTCATTTGTTGTTGGC	TCCTGGCCCTCCTGACCATTACTGAG	(Chico et al., 2018; Wang et al., 2009)
*mhcI*	GACAGTCCGTCCCTCAGTGT	CTGGAAGGTTCCATCATCGT		(Chaves-Pozo et al., 2010)
*mhcII*	TGCCATGCTGATGTGCAG	GTCCCTCAGCCAGGTCACT	CGCCTATGACTTCTACCCCAAACAAAT	(Jorgensen et al., 2009)
*mx1–3*	TGAAGCCCAGGATGAAATGG	TGGCAGGTCGATGAGTGTGA	ACCTCATCAGCCTAGAGATTGGCTCCCC	(Ortega-Villaizan et al., 2011)
*nkef*	CGCTGGACTTCACCTTTGTGT	ACCTCACAACCGATCTTCCTAAAC		(Nombela et al., 2017a)
*vig1*	CTACAATCAAGGTGGTGAACAATGT	GTGGAAACAAAAACCGCACTTATA	TCTCAAGCTTCGGCAACTCCAAGCA	(Nombela et al., 2017a)

### 2.8 Selection of representative rainbow trout immune-related genes

We selected representative genes involved in the innate immune response for further study: major histocompatibility complex I and II (*mhcI*, *mhcII*) and cluster of differentiation 83 (*cd83*) as representative genes for antigen presentation; interferon-induced myxovirus resistant isoforms (*mx1*–*3*), which are important in the rainbow trout response to viral infections (Tafalla et al., 2007); hepcidin (*hep*), an antimicrobial peptide induced by double stranded RNA in rainbow trout (Chiou et al., 2007); interferon-induced protein with tetratricopeptide repeats 5 (*ifit5*) and natural killer enhancing factor (*nkef*), which participate in the antiviral response of rainbow trout RBCs (Chico et al., 2019b; Chico et al., 2021); interferon type 1 (*ifn1*), which is induced by rhabdoviral infections in rainbow trout (Verjan et al., 2008); interleukin 1 beta (*il1b*), a typical pro-inflammatory cytokine in rainbow trout leukocytes (Zou et al., 2000); interleukin 8 (*il8*), a co-modulator of VHSV DNA vaccines in rainbow trout (Jimenez et al., 2006); and virus-induced gene (*vig1*), which has been characterized in VHSV infections in rainbow trout (Boudinot et al., 1999). Table 2 shows the corresponding sequences and references for the primers and probes used.

### 2.9 Statistical analysis

GraphPad Prism 8 was used for statistical analysis and creation of graphics. Statistical tests and associated *P* values were performed for each assay. A clustering heatmap of immune-gene expression data (2 ^−ΔCt^ or 2 ^−ΔΔCt^) was created using Clustvis software (Metsalu and Vilo, 2015). Flow cytometry data were processed and analysed with Flowing Software version 2.5.1 (http://www.flowingsoftware.com). Means and standard deviations were calculated.

## 3 Results

### 3.1 Attachment, uptake, and immune response of rainbow trout RBCs exposed to spinycterins *in vitro*

**Fig. 1:**
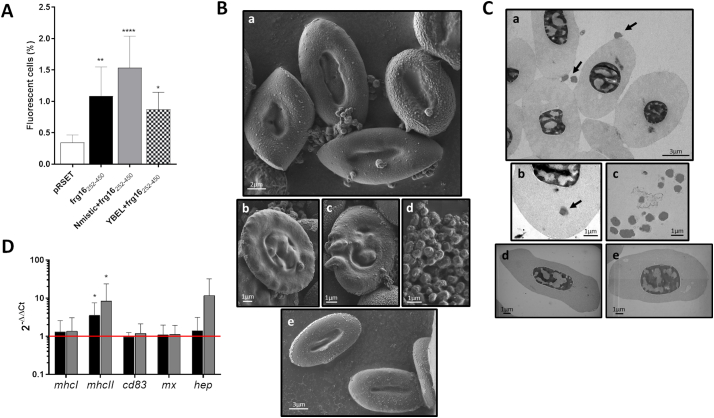
Attachment, uptake, and immune response of rainbow trout RBCs exposed to spinycterins *in vitro*. A) Spinycterins expressing frg16_252–450_, Nmistic+frg16_252–450_, and YBEL +frg16_252–450_ were incubated with rainbow trout RBCs at MOI 40 for 24 h at 14 °C. Spinycterins transformed with the empty pRSET plasmid were included as a control. Mouse anti-polyhistidine antibody and rabbit anti-mouse IgG-FITC antibody were used for fluorescent spinycterin staining. The graph shows the percentage of fluorescent cells calculated by flow cytometry. Data represent means and standard deviations (SD) (*n* = 3). B) Scanning electron microscopy (SEM) micrographs of RBCs treated with Nmistic+frg16_252–450_ spinycterins. Images were taken at the following magnifications: 4 kx (B-a), 7 kx (B-b), 8 kx (B-c), 15 kx (B-d), and 3 kx (B-e). Panel B-a shows spinycterins attached to RBC membranes, and panels B-b and B-c depict protuberances under the RBC membrane that could indicate the presence of spinycterins inside the cells. Panel B-d shows the morphology and size of Nmistic+frg16_252–450_ spinycterins. Panel B-e shows RBCs without spinycterins. C) Scanning transmission electron microscopy (STEM-in-SEM) micrographs of RBCs treated with Nmistic+frg16_252–450_ at MOI 40 for 24 h at 14 °C. Images were taken at the following magnifications: 3.62 kx (C-a), 7.22 kx (C-b), 7.34 kx (C-c), 7.5 kx (C-d), and 9 kx (C-e). The black arrows highlight spinycterins. Panels C-a and C-b show spinycterins attached to the RBC membrane or in the cytosol, and Panel C-c shows the morphology and size of Nmistic+frg16_252–450_ spinycterins. Panels C-d and C-e show RBCs without spinycterins. D) Immune-related gene expression of purified RBCs incubated with spinycterins. Purified RBCs were treated with frg16_252–450_, Nmistic+frg16_252–450_, or empty pRSET plasmid spinycterins for 24 h at 14 °C. Gene expression analysis was performed by qPCR. Data are displayed as mean ± SD (*n* = 8). The *ef1α* gene was used as an endogenous control. Asterisks indicate statistically significance compared to empty pRSET plasmid spinycterins determined by the Kruskal-Wallis test followed by the Dunn multiple comparison method: *,**, and **** indicate *P* < 0.05, *P* < 0.01, and *P* < 0.0001, respectively. The red horizontal bar indicates the control (RBCs treated with empty pRSET plasmid spinycterins). (For interpretation of the references to colour in this figure legend, the reader is referred to the web version of this article.)

We evaluated the attachment of RBCs to spinycterins expressing frg16_252–450_, Nmistic+frg16_252–450_, and YBEL+frg16_252–450_ to predict optimal interactions. Flow cytometry showed thatthe highest RBC-spinycterin interaction was with spinycterins expressing Nmistic+frg16_252–450_ (Fig. 1A). Therefore, spinycterins expressing Nmistic+frg16_252–450_ were compared to those expressing frg16_252–450_ in the remaining experiments to test the role of anchor motifs in the innate immune response of rainbow trout. RBCs exposed to Nmistic+frg16_252–450_ spinycterins *in vitro* for 24 h were visualized by SEM and STEM-in-SEM. SEM images revealed the attachment of Nmistic+frg16_252–450_ spinycterins to the RBC membrane (Fig. 1B-a), with some protuberances under the RBC membrane that could indicate the presence of spinycterins inside the cell (Fig. 1B-b and 1B-c). Fig. 1B-d shows the round morphology and size (500–600 nm) for Nmistic+frg16_252–450_ spinycterins visualized by SEM microscopy, and Fig. 1B-e shows RBCs without spinycterins. STEM-in-SEM images were taken to visualize the uptake or engulfment of spinycterins by RBCs and detected spinycterins on the surface and in the cytosol of RBCs (Fig. 1C-a and 1C-b). Fig. 1C-c shows STEM-in-SEM images of Nmistic+frg16_252–450_ spinycterins, and Figs. 1C-d and 1C-e show RBCs without spinycterins.

To evaluate the immune response of RBCs to spinycterins, purified RBCs were incubated at MOI 40 with frg16_252–450_, Nmistic+frg16_252–450_, or empty pRSET plasmid spinycterins (control) for 24 h at 14 °C*.* Gene expression analysis showed that *mhcII* was significantly upregulated in RBCs treated with frg16_252–450_ and Nmistic+frg16_252–450_ spinycterins compared with control RBCs (Fig. 1D). Upregulation of *hep* expression was detected in RBCs treated with Nmistic+frg16_252–450_ compared to the control, but this difference was not statistically significant.

### 3.2 RBC immune response after intravenous injection of spinycterins

**Fig. 2:**
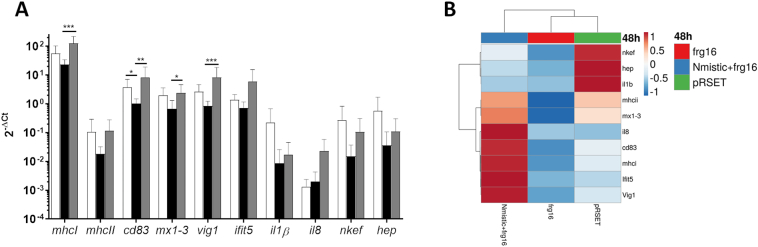
Immune-related gene expression evaluated by qPCR in rainbow trout RBCs isolated from fish intravenously injected with frg16_252–450_ or Nmistic + frg16_252–450_ spinycterins. Rainbow trout RBCs were isolated from blood 48 h after intravenous injection with 480·10^6^ cfu per fish of frg16_252–450_, Nmistic+frg16_252–450_, or control spinycterins (empty pRSET plasmid). A) Gene transcript expression levels of RBCs 48 h after injection. Data are displayed as mean ± SD (*n* = 5). The *ef1α* gene was used as an endogenous control. Statistical differences were analysed by the Kruskal-Wallis test followed by the Dunn multiple comparison method. *, **, and *** indicate *P* < 0.05, *P* < 0.01, and *P* < 0.001, respectively. Black bars indicate frg16_252–450_ spinycterins. Gray bars indicate Nmistic+frg16_252–450_ spinycterins. Empty bars indicate empty pRSET plasmid spinycterins (control). B) The heatmap of molecular signatures (2^−ΔCt^) of RBCs isolated from fish intravenously injected with frg16_252–450,_ Nmistic+frg16_252–450_, or empty pRSET plasmid spinycterins. The dendrogram on top of the heatmap shows clustering of the samples. The heatmap was created using Clustvis software. The heatmap data matrix visualizes the values in the cells using a colour gradient. No data transformation was applied. The columns were collapsed by taking the mean of each group, the rows were centered, and unit variance scaling was applied to rows.

The modulation of innate immune–related genes in rainbow trout circulating RBCs was analysed 48 h after intravenous injection of frg16_252–450_ and Nmistic+frg16_252–450_ spinycterins. We detected upregulation of antigen presentation–related genes (*mhcI*, and *cd83*), interferon-related genes (*vig1* and *ifit5*), and chemokine *il8* in RBCs from fish injected with Nmistic+frg16_252–450_ spinycterins compared to those injected with frg16_252–450_ or empty pRSET plasmid spinycterins (Fig. 2A). The upregulation of *mhcI, cd83, mx1–3,* and *vig1* in RBCs from fish injected with Nmistic+frg16_252–450_ spinycterins compared to frg16_252–450_ spinycterins was statistically significant (Fig. 2A). To evaluate the gene expression profile in response to each treatment, we conducted a multivariate analysis of the gene expression data matrix. The clustering heatmap clearly differentiated a trend in gene expression of RBCs from fish injected with the different spinycterins. The gene expression profile of RBCs from Nmistic+frg16_252–450_ spinycterin–injected individuals markedly contrasted with those injected with empty pRSET plasmid spinycterins (Fig. 2B), and individuals injected with frg16_252–450_ spinycterins showed lower gene expression levels compared with other treatments (Fig. 2B).

### 3.3 Head kidney and spleen immune response after intravenous injection of spinycterins

**Fig. 3:**
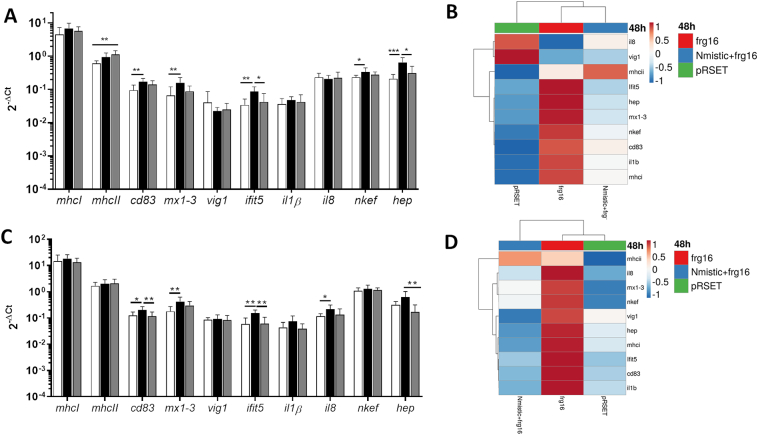
Immune-related gene expression evaluated by qPCR of head kidney and spleen from rainbow trout intravenously injected with frg16_252–450_ or Nmistic + frg16_252–450_ spinycterins. Rainbow trout organs were isolated 48 h after injection with 480·10^6^ cfu per individual of frg16_252–450_, Nmistic+frg16_252–450_, or control (empty pRSET plasmid) spinycterins. A) Gene transcript expression levels in fish head kidney (HK). B) Heatmap of molecular signatures (2^−ΔCt^) in HK 48 h after spinycterin injection. C) Gene transcript expression levels in fish spleen. D) Heatmap of molecular signatures (2^−ΔCt^) of the spleen 48 h after spinycterin injection. Data are displayed as mean ± SD (*n* = 5). The *ef1α* gene was used as an endogenous control. Statistical differences were analysed by the Kruskal-Wallis test followed by the Dunn multiple comparison method. *, **, and *** indicate *P* < 0.05, *P* < 0.01, and *P* < 0.001, respectively. Black bars indicate frg16_252–450_ spinycterins. Gray bars indicate Nmistic+frg16_252–450_ spinycterins. Empty bars indicate empty pRSET plasmid spinycterins (control). The dendrogram on top of the heatmap shows clustering of the samples. Heatmaps were created using Clustvis software. The heatmap data matrix visualizes the values in the cells using a colour gradient. No data transformation was applied. The columns were collapsed by taking the mean of each group, the rows were centered, and unit variance scaling was applied to rows.

The head kidney and spleen are important hematopoietic organs and reservoirs for RBCs in fish (Sahoo et al., 2021; Witeska, 2013). To evaluate the spinycterin-triggered innate immune response in rainbow trout, we analysed the gene transcriptional response in head kidney and spleen 48 h after intravenous injection of spinycterins. Transcriptional analysis of head kidney showed upregulation of antigen presentation–related genes (*mhcI*, *mhcII* and *cd83*), interferon-related genes (*mx1–3* and *ifit5*), *nkef*, and *hep* in individuals injected with frg16_252–450_ or Nmistic+frg16_252–450_ compared to those injected with empty pRSET plasmid spinycterins. Compared to fish injected with empty pRSET plasmid spinycterins, the upregulation of *cd83*, *ifit5*, *mx1-3*, *nkef* and *hep* was statistically significant in fish injected with frg16_252–450_ spinycterins, as was the upregulation of *mhcII* in fish injected with Nmistic+frg16_252–450_ (Fig. 3A). The clustering heatmap clearly differentiated the tendency of frg16_252–450_ spinycterins to upregulate most genes analysed in head kidney compared with Nmistic+frg16_252–450_ or empty pRSET plasmid spinycterins except for *mhcII*, which was upregulated by Nmistic+frg16_252–450_ spinycterins, and *vig1* and *il8*, which were upregulated by pRSET (Fig. 3B).

Transcriptional analysis of the spleen showed significant upregulation of *cd83*, *mx1–3*, *ifit5*, and *il8* in frg16_252–450_ spinycterin–injected individuals compared to those injected with empty pRSET plasmid spinycterins (Fig. 3C). The clustering heatmap of splenic gene expression clearly showed upregulation of most of the analysed genes in fish injected with frg16_252–450_ compared with Nmistic+frg16_252–450_ or empty pRSET plasmid spinycterins, except for *mhcII*, which was upregulated by Nmistic+frg16_252–450_ (Fig. 3D).

### 3.4 Comparison of tissue immune response after intravenous injection of spinycterins

**Fig. 4:**
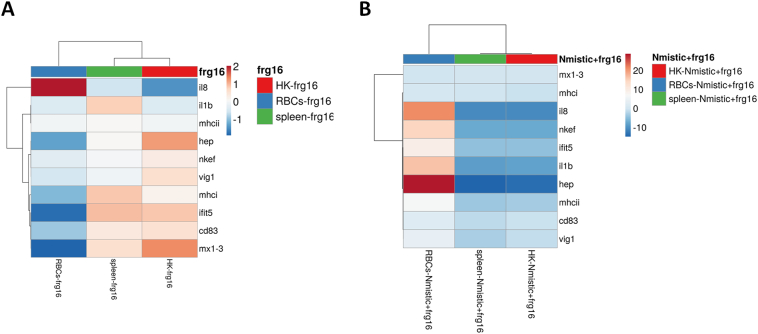
Clustering heatmap of molecular signatures of rainbow trout RBCs, head kidney, and spleen isolated from fish intravenously injected with frg16_252–450_ or Nmistic + frg16_252–450_ spinycterins. Clustering heatmap of molecular signatures (2^−ΔΔCt^) in RBCs, head kidney (HK), and spleen from fish at 48 h after injection of A) frg16_252–450_ or B) Nmistic+frg16_252–450_ spinycterins compared to control (empty pRSET plasmid) spinycterins. The dendrogram on top of the heatmap shows clustering of the samples. Heatmaps were created using Clustvis software. The heatmap data matrix visualizes the values in the cells using a colour gradient. No data transformation was applied. The columns were collapsed by taking the mean of each group, the rows were centered, and unit variance scaling was applied to rows.

We performed multivariate analysis of the gene expression data matrix between the 3 tissues (RBC, head kidney, and spleen) for each spinycterin treatment. The clustering heatmap of fish injected with frg16_252–450_ primarily showed that genes upregulated in the spleen and head kidney (particularly *mx1–3*, and *ifit5*) were downregulated in RBCs. However, *il8* was upregulated in RBCs but downregulated in spleen and head kidney (Fig. 4A). The clustering heatmap of fish injected with Nmistic+frg16_252–450_ showed a similar pattern between spleen and head kidney gene expression. Expression of *il8*, *nkef*, *ifit5*, *il1b*, and *hep* was upregulated in RBCs but downregulated in head kidney and spleen (Fig. 4B). High gene expression levels were induced for most genes tested in RBCs, head kidney, and spleen from fish injected with spinycterins compared with PBS (data not shown).

## 4 Discussion

The role of nucleated RBCs in the immune response has been increasingly realized in recent years. Fish RBCs have membrane receptors for PAMPs (Morera et al., 2011; Morera and Mackenzie, 2011), produce immune-related responses when exposed to or infected by fish viruses (Nombela et al., 2019; Workenhe et al., 2008; Wessel et al., 2015; Wessel et al., 2018; Nombela et al., 2017a; Chico et al., 2019b; Nombela et al., 2017b), can activate pathways to regulate the expression of immune-involved genes such as type I interferon and interferon-stimulated genes (ISGs), can express antigens encoded by a DNA vaccine (Puente-Marin et al., 2018b), can produce cytokines that communicate with and stimulate neighbouring cells (Puente-Marin et al., 2019a; Puente-Marin et al., 2018b), and can mount cellular and humoral immune responses to viral antigens (Puente-Marin et al., 2019a). In the search for new and practical alternatives for prophylaxis in aquaculture, bacterial inclusion bodies encoding viral antigens have shown promise for oral prophylactic delivery (Thwaite et al., 2018). Previously, a study demonstrated that fish RBCs can engulf inclusion bodies made of frg16_252–450_ and modulate innate immune–related genes and proteins in response (Puente-Marin et al., 2019b). In addition, RBCs were found to be able to engulf bacteria and particles *via* different pathways (Sun et al., 2019). In the present study, we introduced a downsized frg16_252–450_ viral fragment into spinycterins as an alternative aquaculture prophylactic method (Coll, 2017). Our results showed that RBCs may play an important role in enhancing the piscine immune response when the antigen is expressed on the surface of spinycterins (Nmistic+frg16_252–450_) and that rainbow trout RBCs responded differently to Nmistic+frg16_252–450_ or frg16_252–450_ spinycterins both *in vitro* and *in vivo*. Although RBCs have been shown to be able to engulf bacteria (Sun et al., 2019) and bacterial inclusion bodies (Puente-Marin et al., 2019b), and modulate an immune response accordingly, our results suggest that spinycterins with membrane-anchored viral antigen might be engulfed and module the RBC innate immune response better than those with the antigen inside.

It is curious that frg16_252–450_ spinycterins caused lower gene expression levels in RBCs than the control group. Downregulation of genes related to type I IFN and antigen presentation–related genes was previously observed in RBCs from rainbow trout intravenously injected with frg16_252–450_ bacterial inclusion bodies at 24 h post injection (Puente-Marin et al., 2019b). It is difficult to know the cause of this lower expression and additional experiments are needed to address that issue, but it seems that at early times post injection, frg16_252–450_ induces downregulation of some genes related to the immune response. However, here we show that when frg16_252–450_ is anchored to the bacterial surface (*i.e.*, Nmistic+frg16_252–450_), the immune response at 48 h after injection is more pronounced, and upregulation of type I IFN and antigen presentation–related genes is observed in RBCs.

Our results confirmed pathways previously described in the rainbow trout RBC response to frg16_252–450_ bacterial inclusion bodies, such as those related to antigen presentation (*mhcI, mhcII*, and *cd83*) and interferon-related genes (*mx1–3, nkef*, *il1b*, and *il8*) (Puente-Marin et al., 2019b). In addition to the expression of genes with direct antiviral activity against VHSV infection (*i.e.*, *mx1–3*, *vig1*, or *ifit5*), spinycterins induced interleukin *il8* activation in rainbow trout RBCs to distribute signals throughout the fish body and trigger additional defensive responses at later times. Similar findings were found for rainbow trout RBCs with a VHSV DNA vaccine (Puente-Marin et al., 2018b) and frg16_252–450_ inclusion bodies (Puente-Marin et al., 2019b).

The head kidney is the major piscine hematopoietic organ in which phagocytosis, antigen processing, and B cell maturation and differentiation occur (Tort et al., 2003). The spleen is known as the primordial secondary lymphoid organ in teleost fish and is responsible for filtering peripheral blood (Bjorgen and Koppang, 2021). The head kidney and spleen demonstrated an immune response when fish were intravenously injected with spinycterins containing different frg16_252–450_ constructs. Previous studies in which recombinant bacterins expressing a viral antigen of infectious spleen and kidney necrosis virus (ISKNV) were injected in fish showed strong upregulation of nonspecific and specific immune-related genes, such as *mx*, in the spleen (Fu et al., 2016). Our results showed increased transcription of interferon-related genes (*mx1–3* and *ifit5*) in response to frg16_252–450_ and Nmistic+frg16_252–450_ spinycterins in the spleen and head kidney, which has been reported in response to VHSV, GVHSV transfection (Tafalla et al., 2007; Hwang et al., 2018), and GVHSV vaccination (Pereiro et al., 2014; Lazarte et al., 2017). In the present work, we showed increased expression of antigen-presenting genes (*mhcI, mhcII*, and *cd83*) in the head kidney and a modest increase in expression of *il1b* in both the head kidney and spleen in response to spinycterins. Increased gene expression has been described after DNA vaccination (Lazarte et al., 2017) and upon VHSV challenge in DNA-vaccinated fish (Cuesta and Tafalla, 2009). Our results show that the response triggered by spinycterins is consistent with the previously reported immune response after VHSV challenge and vaccination.

A multivariate analysis of the gene expression data matrix between RBCs, head kidney, and spleen for each spinycterin treatment revealed a distinct immune response in the organs. In particular, RBCs demonstrated a different immune response than that observed in spleen and head kidney. The clustering heatmap of fish injected with frg16_252–450_ showed that genes upregulated in the spleen and head kidney were downregulated in RBCs and *vice versa*. For example, *il8* was upregulated in RBCs but downregulated in spleen and head kidney. The clustering heatmap of fish injected with Nmistic+frg16_252–450_ showed that the predominant immune response occurred in RBCs compared to spleen and head kidney, mainly for *il8*, *nkef*, *ifit5*, *il1b*, and *hep*. The RBC gene expression profile was seemingly opposite to the profile in spleen and head kidney depending on how the antigen was expressed in the injected spinycterin (intra-spinycterin or surface-anchored). It could be hypothesized that RBCs respond earlier or stronger when directly encountering an antigen on the spinycterin surface, while cells in spleen and head kidney, due to their well-known capacity to phagocytose and present antigens, might generate a better immune response to an antigen expressed within spinycterins. To our knowledge, this is the first report showing that the RBC immune response against a viral antigen exceeds that of well-known immune-involved organs such as the head kidney and spleen. This could indicate that RBCs have a wider role in the early innate immune response that could be especially relevant given the high number of RBCs within the organism. Understanding the responses triggered by vaccines and the potential role of cells involved is essential to develop effective vaccine strategies.

In previous studies, bacterial inclusion bodies made of frg16_252–450_ were able to activate adaptative systemic response (Thwaite et al., 2018; Thwaite et al., 2020). Knowing that fish RBCs are important contributors to the innate and adaptive immune response (Puente-Marin et al., 2019a; Puente-Marin et al., 2018b), the findings presented here and from previous studies support these cells as important vaccine targets. The molecular mechanisms and immune response underlying aquaculture vaccines remain poorly understood. To address this, our objective was to better understand the role of RBCs in response to a new vaccination strategy based on antigen surface–displaying spinycterins. The contribution of RBCs to the host immune response against GVHSV surface–displaying spinycterins, together with the ability of RBCs to recognize and engulf bacteria, process and present antigens, and elicit a humoral response, make RBCs promising target cells for spinycterin-based aquaculture vaccination strategies.

The following are the supplementary data related to this article.

## CRediT authorship contribution statement

**S. Puente-Marin:** Methodology, Validation, Formal analysis, Investigation, Writing – original draft, Visualization. **D. Cazorla:** Methodology, Validation, Formal analysis. **V. Chico:** Validation, Visualization, Writing – review & editing. **J. Coll:** Conceptualization, Supervision, Project administration, Funding acquisition. **M. Ortega-Villaizan:** Conceptualization, Writing – review & editing, Supervision, Project administration, Funding acquisition.

## Declaration of Competing Interest

None.

## Data Availability

Data will be made available on request.
